# Novel Oral Anticoagulants in Patients With Atrial Fibrillation and Moderate to Severe Mitral Stenosis: A Systematic Review

**DOI:** 10.7759/cureus.33222

**Published:** 2023-01-01

**Authors:** Mohamed N Al Rawahi, Juhaina S Al-Maqbali, Jawahar Al Noumani, Abdullah M Al Alawi, Vidal Essebag

**Affiliations:** 1 Medicine, Sultan Qaboos University Hospital, Muscat, OMN; 2 Clinical Pharmacy, Department of Pharmacy, Sultan Qaboos University Hospital, Muscat, OMN; 3 Internal Medicine, Oman Medical Specialty Board, Muscat, OMN; 4 Cardiac Electrophysiology, McGill University, Montréal, CAN

**Keywords:** bleeding risk, septic embolic stroke, atrial fibrillation, direct oral anticoagulant, vitamin k antagonists, mitral stenosis

## Abstract

The use of novel oral anticoagulants (NOAC) in patients with moderate to severe mitral stenosis (MS) and atrial fibrillation (AF) is not recommended. We aimed to evaluate the efficacy and safety of NOAC usage compared to vitamin K antagonist (VKA) in patients with moderate to severe MS and AF. We conducted a systematic review to identify articles that compared warfarin to NOAC in patients with moderate to severe MS and AF. Only four studies (two observational studies and two trials) met our search criteria and reported a total of 7529 patients with MS and AF with MS and AF, 4138 of them treated with NOAC. In both observational studies, the severity of MS was not determined, and there was heterogeneity in MS etiology. Nevertheless, both studies showed a positive signal toward the efficacy and safety of NOAC compared to VKA in this population. A randomized pilot trial (n=40) was done on patients with moderate to severe MS, and it showed further acceptable efficacy and safety for rivaroxaban use. However, a larger randomized controlled trial (n=4531) disclosed that VKA (warfarin) led to a significantly lower rate of a composite of cardiovascular events or mortality than rivaroxaban, without a higher rate of major bleeding but not fatal bleeding. Our systematic review provides exploratory information on NOAC safety and effectiveness in patients with MS; it also discourages using NOACs for patients with moderate to severe MS and supports the current treatment guidelines. However, more dedicated clinical trials evaluating the use of NOACs in moderate to severe MS are underway. They will categorically establish the safety profile and clinical effectiveness of NOAC in this high-risk population.

## Introduction and background

Atrial fibrillation (AF) is the most common sustained arrhythmia, with a prevalence of 0.4% in the general population and 9% in patients above the age of 80 years [1]. Patients with valvular AF, defined as AF accompanying moderate to severe mitral stenosis (MS), or patients with mechanical prosthetic valves, are at high risk of thromboembolism [2]. And it was estimated that the risk of ischemic stroke, transient ischemic attack (TIA), and systemic embolization is increased by two to five times in patients with valvular AF compared to patients with non-valvular AF [3,4]. Without anticoagulation, around 25% of patients with moderate to severe MS could die from systemic embolism [5-7].

Up to 80% of patients with MS and systemic embolism show AF on the ECG. The risk of embolic stroke in patients with MS without evidence of AF, especially in the presence of high-risk features such as enlarged left atrium (LA) (LA volume >60 mL/m^2^) or history of systemic embolism is not negligible. Also, patients with MS and AF and a history of thromboembolism have a recurrence rate of 15 to 40 events per 100 patient months, which is the highest reported rate of thromboembolism in patients with AF [8-12]. This has resulted in vitamin K antagonist (VKA) recommendations in moderate-to-severe MS, even in the absence of AF, in international valve disease guidelines [13,14].

The use of NOAC in patients with moderate to severe MS and AF is not recommended by the American College of Cardiology (ACC), the American Heart Association (AHA), and many other international guidelines (Class III) due to the absence of available and supporting evidence in the literature [15,16]. Therefore, we conducted a systematic review to evaluate the efficacy and safety of NOAC usage in patients with moderate to severe MS and AF.

## Review

Methods

The review protocol was registered at the International Prospective Register of Systematic Reviews (PROSPERO; www.crd.york.ac.uk/prospero/) with registration number CRD42021255325. The study was conducted according to Preferred Reporting Items for Systematic Reviews (PRISMA) 2020 [17].

Searches

Two reviewers (AA, JM) searched Medline via Cochrane Central Register of Controlled Trials (CENTRAL), ClinicalTrials.gov, World Health Organization International Clinical Trials Registry Platform (ICTRP), PubMed, Scopus, gray literature, and published abstracts at major cardiology conferences to identify relevant articles from the date of inception until October 31, 2022. The following search terms were included in isolation and combination: “mitral”, “mitral valve”, “mitral valve disease”, “mitral stenosis", "atrial fibrillation”, AF”,” management”, “anticoagulation”, “anticoagulants”, “Non-vitamin K antagonist oral anticoagulants”, “novel oral anticoagulants”, “direct oral anticoagulant”, “dabigatran”, “apixaban”, “edoxaban”,” rivaroxaban”, “vitamin K antagonists “,” warfarin”, “acenocoumarol”, “phenprocoumon”, and “fluindione” (Table 1).

**Table 1:** Search strategies used on databases and registries CENTRAL: Cochrane Central Register of Controlled Trials; ICTRP: ClinicalTrials.gov, World Health Organization International Clinical Trials Registry Platform

Database	Search strategy
PubMed	((Mitral[Title/Abstract] OR mitral stenosis[Title/Abstract] OR mitral valve[Title/Abstract] OR mitral valve disease[Title/Abstract]) AND (Atrial fibrillation[Title/Abstract] OR atrial fibrillation management[Title/Abstract] OR AF[Title/Abstract])) AND (Anticoagulation[Title/Abstract] OR anticoagulants[Title/Abstract] OR non-vitamin K antagonist oral anticoagulants[Title/Abstract] OR novel oral anticoagulants[Title/Abstract] OR NOAC[Title/Abstract] OR direct oral anticoagulant[Title/Abstract] OR DOAC[Title/Abstract] OR dabigatran[Title/Abstract] OR apixaban[Title/Abstract] OR edoxaban[Title/Abstract] OR rivaroxaban[Title/Abstract] OR vitamin K antagonists[Title/Abstract] OR warfarin[Title/Abstract] OR acenocoumarol[Title/Abstract] OR phenprocoumon [Title/Abstract] OR fluindione.[Title/Abstract])
Clinicaltrials.gov	(Mitral OR mitral stenosis OR mitral valve OR mitral valve disease) AND (Atrial fibrillation OR atrial fibrillation management OR AF) AND (Anticoagulation OR anticoagulants OR non-vitamin K antagonist oral anticoagulants OR novel oral anticoagulants)
CENTRAL	(Anticoagulation OR anticoagulants OR non-vitamin K antagonist oral anticoagulants OR novel oral anticoagulants OR NOAC OR direct oral anticoagulant OR DOAC OR dabigatran OR apixaban OR edoxaban OR rivaroxaban OR vitamin K antagonists OR warfarin OR acenocoumarol OR phenprocoumon OR fluindione.):ti,ab,kw AND (Atrial fibrillation OR atrial fibrillation management OR AF):ti,ab,kw AND (Mitral OR mitral stenosis OR mitral valve OR mitral valve disease):ti,ab,kw (Word variations have been searched)
Scopus	(TITLE-ABS KEY (mitral OR mitral AND stenosis OR mitral AND valve OR mitral AND valve AND disease) AND TITLE-ABS-KEY (atrial AND fibrillation OR atrial AND fibrillation AND management OR AF) AND TITLE-ABS-KEY (anticoagulation OR anticoagulants OR non-vitamin AND k AND antagonist AND oral AND anticoagulants OR novel AND oral AND anticoagulants OR noac OR direct AND oral AND anticoagulant OR doac OR dabigatran OR apixaban OR edoxaban OR rivaroxaban OR vitamin AND k AND antagonists OR warfarin OR acenocoumarol OR phenprocoumon OR fluindione)
ICTRP	(Mitral OR mitral stenosis OR mitral valve OR mitral valve disease) AND (Atrial fibrillation OR atrial fibrillation management OR AF) AND (Anticoagulation OR anticoagulants OR non-vitamin K antagonist oral anticoagulants OR novel oral anticoagulants)

Study Selection

We included all studies, without a language restriction, which compared warfarin to NOAC in patients with moderate to severe MS and AF. Two reviewers (AA, JM, JN, or MR) independently screened titles and abstracts and subsequently performed full-text articles review. A third reviewer (AA, JM, or MR) resolved any reviewer disagreements. In addition, references of eligible studies were screened for additional studies meeting the criteria. The review included all articles published in peer-reviewed journals while review articles, animal studies, case reports, letters, and commentaries were excluded. We used the Covidence platform (https://www.covidence.org) to organize and conduct our systematic review.

Data Extraction

All relevant studies' details, including the year of publication, first author, country, participants’ relevant baseline characteristics (e.g., age, sex, thromboembolism risk, bleeding risk, type and severity of valvular heart disease), follow-up period, study design, inclusion and exclusion criteria, and outcome measures (e.g., stroke, bleeding) were extracted by JM and validated by JN and MR.

Quality Assessment

The quality assessment was performed independently by JM and JN, and any disagreement was resolved by AA. A modified version of the Newcastle-Ottawa scale was used to assess the quality of the included observational studies [18]. The modified Newcastle-Ottawa scale contains eight questions for evaluation of the following: information bias, selection bias, detection bias, performance bias, and comparability of groups of participants (when applicable).

In addition, the modified Cochrane risk of bias scale was used to assess the quality of the included clinical trials. The modified Cochrane risk of bias scale contains five questions for evaluation of the following: sequence generation, allocation concealment, blinding of participants and personnel, complete assessment, and outcome reporting.

Data Synthesis

We narratively reported the findings of the included studies. However, due to the lack of meaningful, relevant data, it was not feasible to perform a metanalysis.

Results

Study Selection

**Figure 1:**
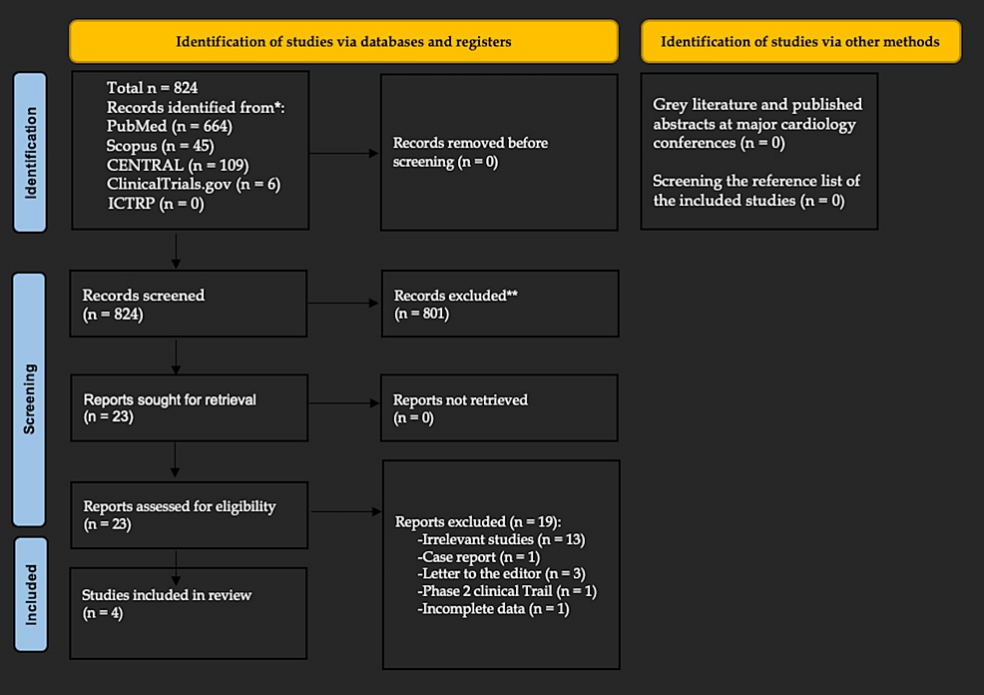
PRISMA flow diagram of study selection PRISMA: Preferred Reporting Items for Systematic Reviews

A total of 824 references were imported for screening. After excluding 801 studies based on duplicates and screening titles and abstracts, the remaining 23 studies were assessed for full-text eligibility. Of these 23 studies, 18 were irrelevant, and an additional study was excluded due to the inability to obtain data related to the severity of MS from the corresponding author [19]. Only four studies were included in this review (Figure 1) [20-22].

Study Characteristics

A total of 7529 patients with MS and AF were included in all the studies; 4138 of them were treated with NOAC. Two retrospective studies, published in 2016 and 2019, reported 2958 patients with MS and AF (1843 of them were treated with NOAC) [20]. Also, one randomized pilot trial, published in 2021, reported 40 patients with MS and AF (20 of them were treated with NOAC). Recently, a randomized control trial (RCT) published in 2022 reported 4531 patients with MS and AF (2275 of them were treated with NOAC); among them 3711 with moderate to severe MS (1871 on rivaroxaban and 1840 on warfarin). This study accounts for 81.9% of the patients (Table 2) [20-23].

**Table 2:** Characteristics of the studies included in the systematic review SD: standard deviation; NSAID: nonsteroidal anti-inflammatory drugs; NOAC: novel anticoagulants, AF: atrial fibrillation; DOAC: direct oral anticoagulant; MS: mitral stenosis, AF: atrial fibrillation; RCT: randomized controlled trial; VKA: vitamin K antagonist

1^st^ author/year	Country	Study design	Main study aim	Assessment period	Total sample size	Age, years	Female n (%)
Noseworthy 2016 [20]	USA	Retrospective cohort (NOAC vs warfarin)	To provide some insight into the effectiveness and safety of NOAC use across the spectrum of valvular heart disease	2010-2015	20,158 patients with valvular AF were on NOAC. Among them 728 had MS, 74 had rheumatics, and 654 had non-rheumatics.	For the MS group (67 - 85 yrs.)	For the MS group (397; 54.5%)
Kim 2019 [21]	Korea	Retrospective cohort (DOAC vs warfarin)	To validate the efficacy of NOACs in patients with mitral stenosis and AF	2008-2017 (Mean follow-up period was 27 months)	2230 patients with AF and native MS. Among them, 1115 were on NOAC and 1115 were on warfarin.	65 -74 yrs.	1548 (69.4%)
Sadeghipour 2022 [22]	Iran	pilot registered RCT	To report efficacy and safety results on the NOAC rivaroxaban patients with AF and moderate-severe MS compare to warfarin	2019-2020	40 patients (AF + mod-severe MS): 20 on rivaroxaban (1 refused anticoagulation), 20 on warfarin	18-75 yrs.	-
Connolly 2022 (INVICTUS) [23]	Africa/Asia/Latin/America (24 countries)	RCT open labeled with blinding to outcomes	To evaluate the efficacy and safety of the factor Xa inhibitor rivaroxaban compared to vitamin K antagonist in patients with rheumatic heart disease with atrial fibrillation (both MS and MR included)	2016-2019	4531 patients with AF and rheumatic heart disease, (2275 on rivaroxaban, and 2256 on warfarin)among them 3711 with moderate-severe MS (1871 on rivaroxaban and 1840 on warfarin)	50.5±14.6 yrs. (mean)	72.3% (3276)

Quality Assessment

All potential sources of bias, including selection, performance, detection, and information, were assessed. As a result, two observational studies were found to have a low risk of bias (Table 3).

**Table 3:** Quality assessment using the modified Newcastle-Ottawa Scale (NOS) for observational studies *1-8 are the elements of the modified Newcastle-Ottawa Scale (NOS) for quality assessment of the studies included in the systematic review. **A score of 0-39% indicates a high risk of bias, 40%-69% a moderate risk of bias, and 70%-100% a low risk of bias.

Study	1	2	3	4	5	6	7	8	Total*	Score**
Noseworthy 2016 [20]	3	3	2	3	1	3	3	1	19/24	79.0%
Kim 2019 [21]	3	3	1	3	2	3	3	3	21/24	87.5%

One clinical trial was found to have a moderate risk of bias. In contrast, the other clinical trial was found to have a low risk of bias (table 4).

**Table 4:** Quality assessment using modified Cochrane risk of bias for clinical trials *1-5 are the elements of Modified Cochrane risk of bias for quality assessment of the clinical trials included in the systematic review. **A score of 0-39% indicates a high risk of bias, 40%-69% moderate risk of bias, and 70%-100% low risk of bias.

Study	1	2	3	4	5	Total*	Score**
Sadeghipour 2022 [22]	3	2	1	2	3	10/15	66.7%
Connolly 2022 (INVICTUS) [23]	3	2	3	3	2	13/15	87.7%

Outcomes

Noseworthy et al. retrospectively analyzed data on the administrative claims of patients with AF who received warfarin, dabigatran, rivaroxaban, or apixaban over five years. The primary effectiveness outcomes were ischemic stroke, hemorrhagic stroke, or systemic embolism while the primary safety outcomes were major bleeding, gastrointestinal bleeding, and other bleeding sites. There were 20,158 patients with valvular heart disease treated with NOAC, including 728 patients with MS of undetermined severity (74 patients had rheumatic MS). Compared to warfarin, patients with MS who were treated with NOAC had a lower risk of stroke (hazard ratio 1 0.52 (0.15-1.81), P =.31) and lower risk of major bleeding (HR 0.77 (0.41-1.43), P =.40). However, these differences were not statistically significant due to the heterogeneity of the cohort and the small sample size; therefore, the data needed to be more conclusive. The retrospective nature of the study has its limitations and biases due to the lack of detail regarding specific valvular pathologies, the severity of valvular stenosis included, and the lack of subgroup analysis [20].

Similarly, Kim et al. retrospectively analyzed a database of patients diagnosed with MS and AF treated with either NOAC or warfarin. The primary efficacy outcome was ischemic stroke or systemic embolization and the safety outcome was intracranial bleeding. A total of 2,230 patients (1,115 received warfarin and 1,115 received NOAC) were included in propensity score matching. Patients with MS treated with NOAC had a lower thromboembolism risk (adjusted HR 0.28 [0.18-0.45]) and lower occurrence of intracranial hemorrhage (adjusted HR 0.53 (0.22-1.26)). Patients were followed up for 27 months, and the overall cumulative incidence curves showed a more significant reduction in ischemic strokes and systemic embolization in the NOAC group compared to the warfarin group ((log-rank P < .0001). While, the rate of intracranial hemorrhages showed a nonsignificant difference between the NOAC group and warfarin group (NOAC group, 0.49%/year; warfarin group, 0.93%/year; adjusted HR: 0.53; 95% CI: 0.22 to 1.26) The Kaplan-Meier curves for the safety outcomes were similar for the NOAC and warfarin groups (log-rank p 1⁄4 0.14) [21]. Although the sample size was big enough to draw a conclusion, the lack of information on the severity of MS and the international normalized ratio (INR) limits the study's validity.

Sadeghipour et al. randomly treated adult patients with an echocardiographic diagnosis of moderate-to-severe MS and AF with a 1:1 ratio to rivaroxaban or warfarin. There were a total of 40 patients (20 received warfarin and 20 received rivaroxaban). The primary outcomes were symptomatic ischemic strokes and systemic embolic events during a 12-month follow-up. The secondary (safety) outcomes were major and clinically relevant nonmajor bleeding. None of the patients developed symptomatic ischemic strokes or systemic embolic events during the 12-month follow-up. However, the rate of silent cerebral ischemia was found in 13.3% (2/15) in the rivaroxaban group and 17.6% (3/17) in the warfarin group. No major bleeding was reported, and only one clinically relevant nonmajor bleeding event (increased menstrual bleeding) was reported in the rivaroxaban group. Despite being a randomized trial for a specific population of moderate-to-severe MS and AF, a significant limitation occurred due to an unpowered sample for primary outcomes. Also, a selection bias was reported, as they excluded severe cases at higher risk for stroke [22].

Lastly, the INVICTUS-VKA (INVestIgation of rheumatiC AF Treatment Using VKAs, rivaroxaban, or aspirin studies) is a prospective, randomized, parallel, open-label clinical trial with a blinded review evaluated the non-inferiority of once-daily rivaroxaban (at a dose of 20 mg or 15 mg versus VKA therapy in patients with documented rheumatic heart disease and AF). They analyzed data for 4531 patients from different sites in 23 countries in Africa, Asia, and South America. Patients with mitral stenosis with a mitral-valve area of no more than 2 cm^2^ were randomized to a 1:1 ratio and were seen in follow-up at one month after randomization and every six months after that with a mean duration of follow-up 3.1±1.2 years). A total of 2292 patients were assigned to the rivaroxaban group, and 2273 to the VKA group, among them 3711 had moderate to severe MS, with valve area ≤2.0 cm^2 ^(1871 on rivaroxaban and 1840 on warfarin), however, the data were presented by the authors as a combined result of all patients without subgroup analysis (Table 5) [23].

**Table 5:** Clinical outcomes in patients with mitral stenosis treated with novel oral anticoagulants compared to patients treated with warfarin NOAC = novel anticoagulants; MS = mitral stenosis; IR = incident rate per 100 person years; HR = hazard ratio; CI: confidence interval; VKA: vitamin K antagonist

Study name		Effectiveness (thromboembolic event)			Safety (major bleed)				
For mitral stenosis		ischemic stroke		systemic embolism	intracranial hemorrhage	gastrointestinal bleeding,		bleeding from other sites	
Noseworthy 2016 [20]	Warfarin	-		-	-		-		-
	NOAC	Combined result for rheumatic + non-rheumatic MS hazard ratio [1] 0.52 [0.15 – 1.81], p = 0.31		-	-		-		-
	Compression (Cox proportional hazards models)	Rheumatic MS (74 patients): NOAC IR = 0 Warfarin IR = 0 HR (95% CI) = NA P = NA Non-rheumatic MS (654 patients)­: NOAC IR = 0.92 Warfarin IR = 1.71 HR (95% CI) = 0.51 (0.15, 1.77) P = 0.29			Combined result for rheumatic + non-rheumatic MS major bleed risk: HR 0.77 [0.41–1.43], p = 0.40 == Rheumatic MS (74 patients): NOAC IR = 3.13 Warfarin IR = 7.53 HR (95% CI) = 0.35 (0.04, 3.33) P = 0.36 Non-rheumatic MS (654 patients)­: NOAC IR = 4.58 Warfarin IR = 5.06 HR (95% CI) = 0.84 (0.44, 1.61) P = 0.60				
Kim 2019 [21]					For Intracranial hemorrhage “only”				
	Warfarin	4.19%/year (146 of 1115 patients)			0.93% (36 of 1115 patients)				
	NOAC	2.22%/year (30 of 1115 patients)			0.49% (7 of 1115 patients)				
	Compression (Cox proportional hazards model)	Adjusted hazard ratio for NOAC: 0.28; 95% confidence interval: 0.18 to 0.45. overall cumulative incidence curves showed a greater reduction in ischemic strokes or systemic embolisms in the NOAC group (survival 98% at 30 months) compared with the warfarin group “survival 90% at 30 months” (log-rank p < 0.0001)			Adjusted hazard ratio for NOAC: 0.53; 95% confidence interval: 0.22 to 1.26. Kaplan-Meier curves for the safety outcomes were similar for the NOAC and warfarin groups (log-rank p ¼ 0.14)				
	The overall survival curve demonstrated a reduction of all-cause death in the NOAC group compared with the warfarin group (log-rank p < 0.0001)								
Sadeghipour 2022 [22]	Rivaroxaban vs warfarin	No symptomatic ischemic strokes/ systemic embolic events during a 12-month follow-up. Rate of silent cerebral ischemia: -13.3% (2/15) in the rivaroxaban group - 17.6% (3/17) in the warfarin group (As per MRI at baseline 6/12 months done for 32 patients); Echocardiographic signs of increased thrombogenicity in the left atrial appendage: -27.3% (3/11 on rivaroxaban) -27.3% (3/11 on warfarin) (As per TEE at baseline 6/12 months done for 22 patients)			*No major bleeding was reported * One clinically relevant nonmajor bleeding event (increased menstrual bleeding) was reported in the rivaroxaban group.				
Connolly 2022 (INVICTUS) [23]	Warfarin/ acenocoumarol: (all N=2256)	48 (0.70% per yr)	10 (0.14% per yr)		7 (0.10% per yr)		-	-	
	Rivaroxaban 20 mg or 15 mg od: (all N=2275)	74 (1.08% per yr)	6 (0.09% per yr)		7 (0.10% per yr)		-	-	
	comparison	560 (8.21% per yr) in the rivaroxaban group and 446 (6.49% per yr) in the vitamin K antagonist group had a stroke, systemic embolism, MI, or death from vascular or unknown cause (HR 1.25 (1.10 to 1.41), P <0.001)			No significant between-group difference in the rate of Hemorrhagic stroke HR (95% CI) 1.00 (0.35 to 2.86) as per intention to treat analysis as per on-treatment analysis: Hemorrhagic stroke: 8 (0.13% per in rivaroxaban), 14 (0.21% per yr in VKA), HR (95% CI) 0.63 (0.26 – 1.50) Major bleed in rivaroxaban 40 (0.67% per yr), 56 (0.83% per yr on VKA), HR (95% CI) 0.76 (0.51 – 1.15)				
	survival time was 1599 days in the rivaroxaban group and 1675 days in the vitamin K antagonist group (difference, −76 days; 95% confidence interval [CI], −121 to −31; P<0.001). included: any of the following: a CHA2DS2VASc score of at least 2 (on a scale from 0 to 9, with higher scores indicating a higher risk of stroke), a mitral-valve area of no more than 2 cm2, left atrial spontaneous echo contrast, or left atrial thrombus. NOTE: At the trial visits, the percentages of patients in the VKA group receiving trial medication (not permanently or temporarily discontinued) were 98.0% at 1 year, 97.7% at 2 years, 97.1% at 3 years, and 96.4% at 4 years; while in the rivaroxaban group, the corresponding percentages were 88.7%, 84.4%, 81.2%, and 79.0%., the mean adherence to rivaroxaban therapy was 83.7 ±16.5%. However, analysis at 5 yrs showed VKA has a better profile for stroke and thromboembolic event prevention, P=0.002.								

The primary-outcome event, including stroke, systemic embolism, myocardial infarction, or death, occurred in 24.6% of patients in the rivaroxaban group compared to 19.7% in the VKA group (proportional HR, 1.25; 95% (CI), 1.10-1.41). While the restricted mean survival time was 1599 days in the rivaroxaban group and 1675 days in the VKA group (difference −76 days; 95% CI, −121 to −31 days; P<0.001 for superiority). There was a higher rate of ischemic stroke and incidence of death in the rivaroxaban group compared to the VKA group due to lower rates of sudden cardiac death and death due to mechanical or pump failure in the VKA group than in the rivaroxaban group. Fortunately, there were no significant differences among the groups in terms of safety profile measured by rates of major bleeding while fatal bleeding was lower in the rivaroxaban group [23]. Interestingly, in this systematic review, we have identified two ongoing trials evaluating the efficacy and safety of NOACs in patients with moderate to severe MS and AF. First, the DAVID-MS (DAbigatran for Stroke Prevention In Atrial Fibrillation in MoDerate or Severe Mitral Stenosis) is a prospective, randomized, open-label study that is expected to enroll 686 patients in Hong Kong and China with moderate to severe MS and AF to either dabigatran (110 or 150 mg twice daily) or VKA, dose-adjusted to achieve an INR of 2-3 [24]. The study is designed to evaluate the non-inferiority of dabigatran, compared with VKA, in preventing the primary outcome of stroke or systemic embolism [24]. Second, the ERTEMIS (Edoxaban in patients with Atrial fibrillation and Mitral stenosis) is a randomized, open-label study currently in phase 2, planned to enroll 240 patients (>19 to 80 years old) with AF and MS from six centers in Korea. Aiming to evaluate the efficacy and safety of Edoxaban versus warfarin over a two-year follow-up period measured by the incidence of Ischemic/hemorrhagic stroke or systemic embolism [25].

Discussion

In this systematic review, we sought to evaluate the available literature on the use of NOACs in patients with moderate to severe MS. Two retrospective studies, one randomized pilot trial, and one open label-RCT with a blinded review met our inclusion criteria. In both observational studies (Noseworthy et al. and Kim et al.), the severity of MS was not determined and there was heterogeneity in MS etiology. Nevertheless, both studies showed a positive signal toward the efficacy and safety of NOAC compared to VKA in this population [20,21]. The randomized pilot trial (Sadeghipour et al.) was done on patients with moderate to severe MS, and it showed further acceptable efficacy and safety for rivaroxaban use [22]. However, Connolly et al. (INVICTUS trial) disclosed that VKA (warfarin) led to a significantly lower rate of a composite of cardiovascular events or mortality than rivaroxaban, without a higher rate of major bleeding but not fatal bleeding. The results of this trial support current guidelines [23]. Of note, the INVICTUS trial accounted for 60% of the total number of patients included in this systematic review (Table 2).

Patients with moderate to severe MS have the highest risk of thromboembolic events among patients with AF [9]. In one-third of the patients with MS, the embolic events occur within a month of the onset of AF diagnosis and two-thirds within one year of follow-up [26]. It is not uncommon to have an embolic event as the first manifestation of MS [6].

In contrast to patients with non-valvular AF, patients with MS appear to have larger thrombi located more frequently at locations outside of the left atrial appendage (LAA), even in the absence of AF [27]. This is likely due to blood stasis in the left atrium and the slow-flow velocity of blood through the stenotic mitral valve coupled with uncoordinated and chaotic left atrial contraction secondary to AF [28].

Patients with moderate to severe MS and AF were not enrolled in the pivotal trials comparing VKAs with the NOACs, likely because these patients are at high risk of thromboembolism. As a result, international guidelines only recommend VKA in these patients [15, 16, 29-31]. All the landmark pivotal trials comparing VKAs with NOACs in AF enrolled patients with 'non-valvular' AF and excluded patients with moderate to severe MS or those with mechanical prosthetic valves [32-35]. There were no randomized trials examining the efficacy of anticoagulation (NOAC) in preventing embolic events in patients with moderate to severe MS until 2022 when the INVICTUS trial studies this category of patients with moderate to severe MS and AF (estimated valve area, <2.0 cm2) [23]. The current recommendations are only developed based on retrospective studies showing a four to 15-fold decrease in the incidence of embolic events with anticoagulation using NOAC in these patients [36,37], and more recently, supports that VKA is still the gold standard with better efficacy and a similar safety profile for patients with moderate to severe MS and AF.

The efficacy of VKA in preventing thromboembolic events in patients with MS and AF can be hindered by the poor quality of anticoagulation therapy and low time in therapeutic range (TTR) in these high-risk patients, more pronounced in developing countries [38]. VKA has many other intrinsic limitations due to its mechanism of action. It prevents the synthesis of coagulation factors II, VII, IX, and X, which can be affected by dietary vitamin K consumption [38]. In addition, VKA has a narrow therapeutic window where patients with an INR of less than 2 have a higher risk of ischemic stroke than those with an INR of more than 2. Granger et al. showed that 75% of patients with major bleeding and intracranial hemorrhage had an INR of less than 3, and 50% of patients with ischemic stroke had an INR of more than 2 [34].

Three out of four included studies in this review had significant methodological limitations (two retrospective and one pilot RCT with a small sample size).In contrast, the INVICTUS trial results represent the most valid findings that support the current guideline recommendation for using VKA in treating patients with AF and moderate to severe MS. Two more ongoing trials evaluate the efficacy and safety of NOACs in patients with moderate to severe MS and AF (DAVID-MS trial and ERTEMIS trial) [24,25].

## Conclusions

Despite the paucity of available studies, our systematic review provides exploratory information on NOAC safety and effectiveness in patients with MS. Our systematic review presents evidence against the use of NOACs for patients with moderate to severe MS and supports the current treatment guidelines. However, more dedicated clinical trials evaluating the use of NOACs in moderate to severe MS are underway and will categorically establish the safety profile and clinical effectiveness of NOAC in this high-risk population.
